# Supplementary material for: Healthcare providers’ experiences and recommendations for antiretroviral therapy adherence in Ghana: A facility-based phenomenological study
Source: PLoS One. 2025 Oct 3;20(10):e0333695. doi: 10.1371/journal.pone.0333695 (PMC12494291; doi:10.1371/journal.pone.0333695)
Supplement: Attachment — Submitted filename: Response to Reviewers.docx [file pone.0333695.s003.docx]

**Response to Reviewers’ comments**

| **Comment** | **Response** |
| --- | --- |
| **Reviewer 1:** | |
| Line 64- Can the authors use the more updated UNAIDS 2024 HIV fact sheet for the global HIV statistics. | The UNAIDS 2024 HIV fact sheet has been used to update the statistics as suggested by the reviewer. Kindly refer to page 3 (line 64-65). |
| Figure 1- For consistency and clarity, can the authors consider changing caregiver’s perspectives to “healthcare provider” perspectives in the conceptual framework. | The change has been made in the framework as suggested by the reviewer. Kindly refer to page 4 (figure 1). |
| The aims of the study lack clarity regarding the study population. Was the focus on healthcare workers' perceptions of ART adherence limited to adult patients only, or did it include all populations (children, adolescents, and adults)?" | The study’s population has been clarified as suggested by the reviewer. Kindly refer to page 4 (line 109-114). |
| Although the literature on health care worker perspectives on ART adherence is scarce, it will be useful to review some existing literature to strengthen the study justification. You can consider adding these two articles: Lahai M, Theobald S, Wurie HR, Lakoh S, Erah PO, Samai M, Raven J. Factors influencing adherence to antiretroviral therapy from the experience of people living with HIV and their healthcare providers in Sierra Leone: a qualitative study. BMC Health Serv Res. 2022 Nov 8;22(1):1327. doi: 10.1186/s12913-022-08606-x Moucheraud C, Stern AF, Ahearn C, Ismail A, Nsubuga-Nyombi T, Ngonyani MM, Mvungi J, Ssensamba J. Barriers to HIV Treatment Adherence: A Qualitative Study of Discrepancies Between Perceptions of Patients and Health Providers in Tanzania and Uganda. AIDS Patient Care STDS. 2019 Sep;33(9):406-413. doi: 10.1089/apc.2019.0053. PMID: 31517526; PMCID: PMC6745526. | We have reviewed literature on healthcare workers’ perspectives as suggested by the reviewer. Kindly refer to pape 3 & 4 (line 88-100). |
| Typo in line 122- change “ast” to East | The mistake has been corrected. Kindly refer to page 5 (line 133). |
| Can the authors please specify whether the analysis was deductive or inductive | The analysis was inductive. This has been explained in page 7 (line 2020-204). |
| Ethics- A more detailed discussion on how participant confidentiality and anonymity was maintained needs to be included in the ethics section. | How participants’confidentiality and anonymity were ensured has been explained in page 7 (line 194-198). |
| Recruitment process- The recruitment process is unclear in terms of the number and type of healthcare workers that were targeted for the study. Furthermore, it is unclear why only 18 interviews were conducted. Was data collection stopped once saturation was reached? | The recruitment process has been explained, as well as why 18 interviews were conducted have been duly explained in page 6 ( line 163-175) |
| Line 163- Please change the number 3 in bracket to 2. | The number 3 in the bracket has been changed to 2 as suggested by the reviewer. Kindly refer to page 6 (line 181). |
| The authors have provided comprehensive data. To add depth to their narrative and strengthen the findings, it would be useful if they could also present any negative or outlier cases related to the identified challenges. | The authors appreciate the tangent that the reviewer has suggested to improve the paper. However, upon combing through our transcript, we could not find any data on that. However, we have grouped the experiences of healthcare providers into positive and negative experiences. Kindly refer to page 12 (Table 2). |
| It would also be useful to provide a summary table that helps to better align the study recommendations to the reasons for interrupting ART. This could be done in a tabular format, where you have a column for the challenge, a second column describing the challenge, with a corresponding quote, and a third column with recommendations. | This has been provided in Table 2 (page 11-20) |
| Line 548, the sentence is incomplete | The sentence has been completed. Kindly refer to page 32 (line 61-67). |
| Line 560- There is a repeated word “experiences. Please delete the extra word. | The extra word has been deleted as suggested by the reviewer. Kindly refer to page 32 (line 613-614). |
| Line 593- Please add the word “interruption” after ART | The word “interruption has been added as suggested by the reviewer. Kindly refer to page 33 (line 655). |
| The authors have listed some study limitations. Are there some strengths of the study that was conducted | We have provided some strengths of the study as suggested by the reviewer. Kindly refer to page 38 (lines 796-805). |
| The conclusion is somewhat unclear. It would be beneficial if the authors could emphasize two or three main challenges and provide corresponding recommendations as a take-home message. | The conclusion has been revised as suggested by the reviewer. Kindly refer to page 38 (line 807-904). |
| **Reviewer 2** | |
| Methods Section: Provide more detail on the sampling strategy, data saturation, and justification for the sample size. | Details have been provided on the sampling strategy, data saturation and the sample size as suggested by the reviewer. Kindly refer to page 6 (lines 163-175). |
| The manuscript mentions recruiting healthcare providers with ≥2 years of experience, which aligns with the study objectives. However, it lacks details about data saturation in interviews. | Details on data saturation has been provided on page 6 as suggested by the reviewer (lines 163-175). |
| Limitations: Acknowledge methodological constraints like potential interviewer bias or regional contextual factors. | The limitation section has been revised as suggested by the reviewer. Kindly refer to page 38 (lines 796-805). |
| The authors mention sample size and geographic scope as limitations. but a more comprehensive limitations section (e.g., interviewer bias, and generalizability of qualitative findings) would strengthen the manuscript. | The limitation section has been revised as suggested by the reviewer. Kindly refer to page 38 (lines 796-805). |
| Supplement with Appendices: Provide the interview guide and coding framework in appendices for transparency | The interview guide has been attached as an appendix, as suggested by the reviewer. However, we are of the opinion that the detailed reporting in Table 2 may not warrant the attachment of a coding tree. |
