# Supplementary material for: Healthcare providers’ experiences and recommendations for antiretroviral therapy adherence in Ghana: A facility-based phenomenological study
Source: PLoS One. 2025 Oct 3;20(10):e0333695. doi: 10.1371/journal.pone.0333695 (PMC12494291; doi:10.1371/journal.pone.0333695)
Supplement: Attachment — Submitted filename: Ghana_HIV_review.docx [file pone.0333695.s002.docx]

Dear Authors,

Contradiction for coming up with this policy feeding study in Ghana. Your study design and findings were well narrated and discussed with practical and feasible recommendations to HIV program in the country.

You may consider a few of the following comments.

Line# 69: “ 95-95-95’’ Need to be revised as : ‘’At least 95% of people living with HIV know their HIV status, at least 95% of people who know their HIV status are on treatment, and at least 95% of people on treatment have a suppressed viral load”

Wasn’t it due to saturation reached at 18th participants that 18 health care were selected for the study (line #174)? Line # 188-189 seems to have another justification for 18 participants. Recheck these two sentences, please.

Check if secluded interview can be retained at line#187 or line # 200., to avoid repetition of same idea. Good to mention both but you may remain only under ethical issue.

Please check if the statements under **rigor** might need references. E.g look at statements under lines#227-230

In lines#233-236 please try review and avoid repeated words such as ‘majority, most’. You may put only the %.

Check the way you put the quotation mark under ‘Improvement in adherence to treatment’ and ‘stigmatization’.

Line # 640: Good to suggest support from the government or NGOs. ART is to be taken for life where income generating activities (IGA) might be considered for sustained income.

There have been a lot of PHIV perspective on ART adherence so far in Ghana (line 101), check if PLHIV perspective should be mentioned as limitation (line# 799).
