# Supplementary material for: Healthcare providers’ experiences and recommendations for antiretroviral therapy adherence in Ghana: A facility-based phenomenological study
Source: PLoS One. 2025 Oct 3;20(10):e0333695. doi: 10.1371/journal.pone.0333695 (PMC12494291; doi:10.1371/journal.pone.0333695)
Supplement: Attachment — Submitted filename: REVIEW COMMENTS 2.docx [file pone.0333695.s004.docx]

**Response to authors comments**

Your study design and findings were well narrated and discussed with practical and feasible recommendations to HIV program in the country.

**Response:** Thank you for your comments.

You may consider a few of the following comments.

1. Line# 69: “ 95-95-95’’ Need to be revised as : ‘’At least 95% of people living with HIV know their HIV status, at least 95% of people who know their HIV status are on treatment, and at least 95% of people on treatment have a suppressed viral load”

**Response**: The sentence has been revised as suggested by the reviewer. Kindly refer to line #69-71.

2. Wasn’t it due to saturation reached at 18th participants that 18 health care were selected for the study (line #174)? Line # 188-189 seems to have another justification for 18 participants. Recheck these two sentences, please.

**Response**: This is a mishap. Sample size was based on data saturation as mentioned in line 174. the contradiction in line 188-189 has been deleted.

3. Check if secluded interview can be retained at line#187 or line # 200., to avoid repetition of same idea. Good to mention both but you may remain only under ethical issue.

**Response:** Lines 190-191 has been deleted from the work to avoid the repetition. It has only been retained under the ethical issue in line 200-201.

4. Please check if the statements under **rigor** might need references. E.g look at statements under lines#227-230.

**Response**: A reference has been provided for the section as suggested by the reviewer. Kindly refer to lines 220 and 228.

5. In lines#233-236 please try review and avoid repeated words such as ‘majority, most’. You may put only the %.

**Response**: Lines 234-237 has been revised. Repetition of words such has “majority” and “most” has been deleted.

6. Check the way you put the quotation mark under ‘Improvement in adherence to treatment’ and ‘stigmatization’.

**Response**: The sentence has been revise by removing the quotation marks in the middle of the quote. Kindly refer to Table 2. and line 301.

7. Line # 640: Good to suggest support from the government or NGOs. ART is to be taken for life where income generating activities (IGA) might be considered for sustained income.

**Response.** The paragraph has been revise to incorporate the idea suggested by the reviewer. Kindly refer to lines 648-649.

8. There have been a lot of PHIV perspective on ART adherence so far in Ghana (line 101), check if PLHIV perspective should be mentioned as limitation (line# 799).

**Response:** The mention of perspectives of PLHIV as a limitation has been expunged from the study as suggested by the reviewer. Kindly refer to lines 806-813.
